# Supplementary material for: Are carbon emissions trading and green financial instruments synergistic? -Comprehensive quantitative research based on content analysis
Source: PLoS One. 2024 Mar 7;19(3):e0298601. doi: 10.1371/journal.pone.0298601 (PMC10919601; doi:10.1371/journal.pone.0298601)
Supplement: S1 Data — (ZIP) [file pone.0298601.s001.zip › Data/地级市Z_F工作报告.zip!/░▓╗╒/┴∙░▓╩╨/└┤╫╘╣1⁄2╓┌║┼í╛┬φ┐╦╩2╛▌═°í┐_2014.docx]

各位代表：
　　现在，我代表市人民政府，向大会报告政府工作，请予审议，并请市政协委员和其他列席人员提出意见。
　　一、2013年和本届政府工作回顾
　　2013年，在市委的坚强领导下，我们认真贯彻党的十八大和十八届三中全会精神，牢牢把握稳中求进的工作总基调，凝心聚力，迎难克坚，全市呈现出经济发展、民生改善、社会和谐的良好态势。预计，生产总值增长9%，财政收入增长13.5%。一年来，主要做了以下八个方面工作：
　　（一）稳增长，加大政策落实力度。及时落实并出台稳增长的一系列政策措施，加大对实体经济和小微企业帮扶力度，全年减免缓各项税费16亿元，安排财政专项资金5亿元，预计规模工业增加值增长12%，新增规模企业179户、总数达到949户，其中亿元企业401户、10亿元企业19户。坚持经济工作项目化、项目工作责任化，强力实施市重点项目“253”工程，预计完成固定资产投资838亿元，增长22%。首矿大昌300万吨钢铁、华电六安电厂二期、华润啤酒/水饮品双百万吨、武钢霍邱冶金工业园等项目扎实推进。大力开展产业链招商、精准招商、园区共建招商，加强与央企、知名民企对接，成功承办中国光彩事业六安行活动、第四届中国（六安）国际羽绒博览会，预计招商到位内资830亿元、外资3亿美元，分别增长17.5%、16.8%。落实促进消费和稳定外贸增长政策，预计实现社会消费品零售总额434亿元，增长13.5%；进出口总额8亿美元，增长8.7%。加强银企对接，新增贷款136.1亿元，增长19.8%，增量和增幅均创历史最好水平。住房公积金个人贷款35亿元、项目贷款4亿元。直接融资23.9亿元，获批发行2013年城投债16亿元。
　　（二）夯基础，大力发展现代农业。扎实推进粮食生产“三大行动”，实现粮食总产88.8亿斤。组织开展超级杂交稻攻关试验，水稻单产和百亩连片平均亩产创全省最高水平。深入实施农产品加工“168”工程，预计加工产值突破700亿元，增长14.5%。舒城县、霍山县被授予全国农产品加工示范基地。 全面实施茶产业振兴工程，实现茶叶综合产值48亿元。扎实推进百万亩油茶产业发展行动计划，荣获全国油茶产业发展示范市。积极培育农村新型经营主体，新增农民专业合作社2177家、家庭农场145个，流转土地和规模经营244.6万亩，金安区圆满完成全国农村集体土地承包经营权确权登记试点。大力发展品牌农业，全市农业“三品”认证总数达204个，六安瓜片、霍山石斛入选央视“2013中国十大最具魅力农产品”，霍山灵芝、金寨板栗获国家地理标志产品保护。切实增强农业保障能力，完成农田水利建设投资11.5亿元，裕安区连续三年获全省农田水利基本建设“江淮杯”一等奖。
　　（三）调结构，加快转变发展方式。大力推进技术创新体系建设，新认定省级创新型企业4家、工程技术研究中心3家、知识产权优势企业3家，袁隆平院士亿牛工作站和博微长安国家级企业技术中心挂牌，填补了全市空白；申请发明专利824件、授权124件，分别增长84%、148%，荣获全国科技进步先进市。大力发展高新技术产业，新认定国家高新技术企业20家、省级高新技术产品105个，预计实现产值307亿元，增长14.5%。开发园区转型升级步伐加快，市开发区升级为国家级开发区，市示范园区发展框架全面拉开。扎实推进商标品牌战略，新增中国驰名商标2件、安徽名牌产品17个、安徽著名商标41件。完成旅游业发展第二个三年行动计划，全年共接待游客2000万人次，实现总收入130亿元，分别增长33.3%、36.8%。加强节能减排，实施节能技改项目24个、污染减排项目68个。开展闲置土地清理，大力推进节约集约用地，霍山县荣获全国国土资源节约集约利用模范县。积极推进生态市建设，实施“森林增长工程”43.2万亩，创建国家级生态乡镇1个、省级生态乡镇10个、生态村7个，霍山县成为我省首个全国生态文明建设试点县，舒城县创建国家生态县工作全面启动。
　　（四）促改革，释放体制机制活力。深化行政审批制度改革，实行基本建设项目联合审批和收费“一表制”。进一步理顺政府投融资平台管理体制，积极推进行政事业单位国有资产管理改革。强化规划及土地集中统一管理，城市重点工程建设管理体制不断完善。金寨农村金融改革扎实推进，金融组织体系、信用体系和服务水平取得突破性进展；六安农村商业银行获批筹建，5家村镇银行开业运营。建立综合治税平台信息系统，“营改增”扩围试点改革有序进行。全面开展清理“吃空饷”和市直单位编外聘用人员管理工作，启动工商质检和食品药品监管体制改革。深化城区基础教育改革，稳妥推进城区高中招生制度改革。巩固基层医药卫生体制改革成果，全面推进县级公立医院综合改革。
　　（五）统城乡，提升区域承载能力。完成第二轮中心城市建设重点工程三年实施计划，当年实施7大类91项重点工程建设，完成政府性投资42亿元，苏大堰和大雁河综合治理、残疾人康复中心等项目快速推进，其中亚锦赛场馆、城北橡胶坝等47个项目建成使用。开展征迁拆违百日会战，全年征迁面积206万平方米、是前三年的总和，拆违16.87万平方米。加快东部产业新城建设，调整管理体制，完善运行机制；完成西部生态新区总体规划编制，凤凰河综合治理一期工程完工。深入开展 “五城同创”和国家森林城市创建工作，成功创建省级卫生城市。城乡精神文明建设取得积极成效，霍山县荣获“全国文明县城”称号。推进县城总规修编，城镇建设步伐加快。全面实施美好乡村首批231个中心村特别是83个重点示范村建设，5个乡村入选全国“美丽乡村”创建试点。启动“三线三边一点一地”城乡环境综合治理。开工建设国省干线公路11条、总里程644.7公里，改造县乡道路646公里、危桥102座，建设城市公交换乘中心和首末站10个。强力实施金寨“5+1”项目，大别山职业学校和旅游扶贫快速通道加快建设。扎实推进新一轮扶贫，实施“整村推进”196个。深入推进皖江示范区建设，加快融入合肥经济圈，寿县、霍邱、舒城与合肥市结对合作不断深化。
　　（六）添活力，全面发展社会事业。积极开展中国梦教育活动，深入践行社会主义核心价值体系，张仁强、张仁秀兄妹荣获第四届全国道德模范提名奖。成功举办第二届中国大别山（六安）山水文化旅游节暨首届海峡两岸“十大华语电影”颁奖盛典。寿县荣获“中国书法之乡”，霍山县荣获全省文学创作先进县。实施重大文化产业项目带动，预计实现文化产业增加值47亿元、增长10%，新桥国际阳光半岛、悠然蓝溪、保利文化产业园建设扎实推进。加快实施文化民生工程，公共文化场馆全部免费开放，选择12个村开展农民文化乐园建设试点。提前完成学前教育三年行动计划，完成义务教育标准化学校建设396所，新增省级示范普通高中3所。依托六安职业技术学院组建安徽大别山职业教育集团和裕安分院。进一步健全城乡医疗卫生服务体系，市中医院通过三级甲等中医院评审，新批准民营医疗机构2家。广泛开展全民健身运动，成功举办第十五届亚洲赛艇锦标赛。着力稳定低生育水平，人口自然增长率7.2‰。国防动员和民兵预备役工作扎实推进，第四次荣获全省双拥模范城。人防、档案、民族宗教、外事侨务、对台、气象、地方志、防震减灾、老龄、妇女儿童、新闻出版等各项事业取得新进展。
　　（七）惠民生，维护社会和谐稳定。在实施省33项民生工程基础上，新增实施小街巷综合整治、平价商店建设、城市公交基础设施建设、城市一卡通4项市级项目，累计投入财政资金64亿元。稳定和扩大就业，城镇新增就业4.76万人，城镇登记失业率3.64%。企业退休人员养老金、农村居民最低生活保障、农村五保供养补助和重度残疾人生活救助标准稳步提高。开工建设各类保障性住房2.73万套，基本建成1.84万套，超额完成省政府下达任务，长安厂、三里街地块棚户区改造全面启动。全面加强和创新社会治理，被评为全省社会管理综合治理先进市，霍山县再获全国综治最高奖“长安杯”。健全有效化解社会矛盾机制，深入开展领导干部开门接访、带案下访、包案处访和网上信访活动，认真解决群众反映强烈的突出问题，全市信访形势稳定可控。持续开展“安全生产年”活动，加大食品药品安全监管力度，加快数字化社会治安防控体系建设，严厉打击各种违法犯罪活动，人民群众安全感不断提升。
　　（八）转作风，切实提高行政效能。认真贯彻落实中央八项规定，切实改进作风，提高政府工作的执行力和落实力。深入开展“六五”普法，完善政府议事制度和决策咨询机制。自觉接受人大监督，依法执行人大决议决定，认真办理《关于加快农村电网改造提高供电质量的议案》、《关于进一步加强物业管理的议案》等人大代表议案和建议96件。主动接受政协民主监督，积极吸纳“关于加快工业园区转型发展”等调研成果，办理政协提案215件。密切与工会、共青团、妇联等人民团体的联系。推进招投标、公共资源交易等规范化管理，建立基本建设项目联合审批平台。加快转变政府职能，全面清理市级行政审批项目和规范性文件，审批项目精简率81%，保留项目全省最少，废止规范性文件97件。深入开展“社会评窗口、万人评科长”活动，机关效能建设进一步加强。积极回应网络问政，市政府网站在2013年中国政府网站绩效评估中位列地市级政府网站第15名。强化重点领域、关键环节的行政监察，加强和改进政府投资项目、财政预算执行、领导干部经济责任及其他专项资金审计。加强廉政建设，全面推进廉政风险防控，严控“三公”经费支出，政风建设取得新成效。
　　各位代表，2013年是本届政府任期的最后一年。四年来，我们沉着应对形势变化，积极抢抓难得机遇，团结拼搏，砥砺奋进，开创了幸福六安建设新局面。综合实力跃上新的台阶。预计全市生产总值由564亿元增至1004亿元，年均增长11.5%；财政收入由52.1亿元增至127.7亿元，年均增长25.1%；规模工业增加值由187.1亿元增至400亿元，年均增长20.4%；农业总产值由249.1亿元增至372.4亿元，年均增长4.5%，农业产业化发展水平综合排名由全省第12位上升至第3位。转型升级迈出坚实步伐。三次产业结构由23.5:42.3:34.2调整为20.6:46.9:32.5。工业化率由33%提高到39.5%。金融、旅游、物流等现代服务业蓬勃发展。节能减排任务全面完成，万元GDP能耗累计下降15.6%，提前完成“十二五”淘汰落后产能计划。生态环境得到有效保护，森林资源稳步增长。城乡面貌发生深刻变化。累计完成固定资产投资2583亿元，建成一大批打基础、利长远、惠民生的重大项目。中心城市建成区面积扩展到67.5平方公里，人口增加到66.2万人，荣获“国家园林城市”等称号。城镇建设加快推进，城镇化率达到40%以上。新增铁路营运里程100公里、高速公路通车里程163公里，新建改建国省干道209公里、县乡公路1022公里。治淮3项骨干工程和重点地质灾害点治理全面完成。电力、通讯、网络保障能力明显增强。改革开放取得重大进展。行政审批制度改革深入推进，人事制度改革稳步实施，政府投融资和财税体制改革取得新成效，农村综合改革向纵深推进，文化体制改革连续三年荣获全国先进地区称号。非公有制经济蓬勃发展，比重由61.7%提高到67 %，实现税收由27.3亿元增至89.3亿元。积极承接产业转移，累计招商到位内资2441.4亿元、外资9.8亿美元，年均增长44%、22.8%；累计实现进出口总额26.2亿美元，年均增长24.3%。人民生活得到持续改善。累计投入民生支出211亿元，惠及710万城乡居民。预计城镇居民人均可支配收入21306元，年均增长12%；农民人均纯收入7320元，年均增长16.2%。城乡居民最低生活保障实现应保尽保，五保集中供养率达38.7%；城乡居民养老保险、医疗保险实现全覆盖，基础养老金发放率100%。累计建设各类保障性住房和棚户区改造安置住房9.2万套，改造农村危房7.59万户。公共教育、文化和医疗服务体系不断完善，人民群众安全感指数达到92.88%，社会保持和谐稳定。
　　各位代表，回首过去四年，我们有着太多感慨和激动。这四年，吴邦国、温家宝等中央领导先后亲临六安视察指导，对老区发展作出重要指示，给予我们特别厚爱和极大鼓舞；这四年，党中央、国务院先后出台促进中部崛起、皖江城市带承接产业转移示范区、大别山片区扶贫开发等重大战略规划，把六安纳入规划范围，给予我们特殊帮助和大力支持；这四年，省委、省政府先后作出推进合肥经济圈建设、加快皖北及沿淮部分市县发展、促进大别山革命老区又好又快发展、抓金寨促全省扶贫开发等重要决策部署，为我们加快发展赢得了良好条件、奠定了坚实基础。
　　各位代表，今日六安正处在积聚实力、充满活力、彰显魅力的重要发展阶段。成绩来之不易，未来充满希望。这是省委、省政府和市委正确领导的结果，是全市人民团结奋斗、开拓进取的结果，是历届班子坚持不懈、奋力拼搏的结果。在此，我代表市人民政府，向全市各族人民、各界人士表示崇高的敬意和衷心的感谢!
　　在肯定成绩的同时，我们也清醒认识到经济社会发展中还存在不少困难和问题：发展不足、发展不优、发展不平衡的矛盾还比较突出；产业结构不尽合理，主导产业实力不强，现代服务业发展滞后；经济下行压力较大，去年生产总值、财政收入、规模工业增加值、社会消费品零售总额、进出口总额增幅没有达到预期目标；基本公共服务体系还不健全，社会建设和民生改善方面还有大量工作要做；政府职能转变还不到位，“四风”问题还不同程度存在，行政效能和绩效管理水平有待进一步提高。对此，我们一定高度重视，采取更加有力措施加以解决。
　　二、新一届政府的奋斗目标和主要任务
　　今后四年，是我们抢抓战略机遇、加速跨越崛起、建设幸福六安的关键时期。政府工作的指导思想是：全面贯彻落实党的十八大和十八届三中全会精神，紧紧围绕“五位一体”总体布局，牢牢把握“四化同步”战略部署，以绿色发展为总取向，坚持稳中求进、好中求快，致力提速增效、争先进位，把改革创新贯穿于经济社会发展各个领域和各个环节，更加注重平稳较快增长，更加注重转变发展方式，更加注重改善民计民生，更加注重建设生态文明，更加注重社会和谐稳定，全力加快幸福六安建设进程。
　　根据市第三次党代会部署，今后四年政府工作的主要目标是：
　　——推进实力六安建设。到2017年，全市生产总值达到1500亿元，年均增长10.5%；财政收入达到200亿元，年均增长11.5%；粮食总产达到100亿斤；固定资产投资达到1600亿元，年均增长18%；规模工业增加值达到650亿元，年均增长13%；社会消费品零售总额达到710亿元，年均增长13%。全市综合实力明显增强，质量效益不断提升，产业体系逐步健全。
　　——推进活力六安建设。经济体制改革全面深化，社会、文化、生态文明体制改革取得实质性进展。资本、人才等生产要素加速汇聚，科技创新活力增强，民营经济快速发展。对外开放广度和深度不断拓展，到2017年，全市到位内资达到1300亿元，年均增长12%；利用外商直接投资达到5.2亿美元，年均增长15%；进出口总额达到12.4亿美元，年均增长12%。
　　——推进魅力六安建设。青山绿水得到有效保护和利用，生态环境更加优美。中心城市滨水生态文化特色日益彰显，知名度和美誉度全面提升；新型城镇化步伐加快，美好乡村建设扎实推进，到2017年，全市城镇化率达到46.5%。文化强市建设迈出新步伐，市民文明素质明显提高，文化软实力和影响力持续提升。
　　——推进和谐六安建设。民主法制建设全面加强，社会公平正义得到有效维护。区域、城乡差距逐步缩小，贫困发生率控制在10%以下，人口自然增长率控制在7.6‰以内。群众利益诉求渠道畅通，社会各方利益关系得到妥善协调。社会治理方式进一步改进，公共安全体系进一步健全，社会保持和谐稳定。
　　——推进幸福六安建设。城乡居民收入稳步增长，到2017年分别达到3.12万元、1.15万元，年均分别增长10%、12%，群众生活水平明显提高。民生工程全面加强，事关群众切身利益的实际问题得到有效解决。就业更加充分，社会保障更加完善。基本公共服务体系逐步完备，人民群众幸福指数全面提升。
实现上述目标，今后四年的主要任务：
　　第一，全面深化改革开放。发挥市场在资源配置中的决定性作用，深化国有企业改革，积极发展混合所有制经济，大力发展民营经济，进一步激活各类市场主体活力。深化行政体制改革，积极稳妥实施大部门制改革，创新行政管理方式，进一步转变政府职能；坚持政事分开、政社分开，加快推进事业单位分类改革，培育社会组织。大力推进投融资、财税金融、土地管理、招投标、户籍管理制度改革，建立健全统一开放的资本、技术、人力资源等各类要素市场。深化农村综合改革，加快构建新型农业经营体系。全面深化开放合作，更大规模、更高质量地承接产业转移。主动融入合肥经济圈，深入推进与合肥市在基础设施建设、产业发展、园区合作、生态保护、市场开拓、人才交流等方面合作。鼓励企业“走出去”，提升开拓国际市场能力。
　　第二，打造六安经济升级版。坚持调整存量、优化增量，提升传统产业，培育壮大高新技术产业和战略性新兴产业，大力发展现代服务业，努力构建重点突出、特色明显、更具竞争力的现代产业体系。进一步增强自主创新能力，突出企业主体地位，全面推进制度创新、科技创新、管理创新，加速科技成果转化。加快实施农产品加工“168”工程，打造千亿元产业，进一步提升农业产业化水平。着力改善消费环境，释放居民消费潜力，提升消费对经济增长的贡献。坚持以人的城镇化为核心，进一步提升中心城市功能，加快舒城、霍邱、寿县县城建设中等城市，推动霍山、金寨县城和叶集城区产城一体发展，打造特色集镇，建设美好乡村；到2017年，中心城市建成区面积85平方公里、人口85万人，全市半数以上中心村达到美好乡村建设要求。加快信息化建设，推进信息化应用，“数字六安、智慧城市”框架基本建成。
　　第三，推动文化大发展大繁荣。高举中国梦精神旗帜，扎实推进社会主义核心价值体系建设，大力弘扬新时期六安精神。深入实施公民道德建设工程，广泛开展精神文明创建活动。坚持社会效益和经济效益相统一，进一步深化文化体制改革。积极构建现代文化产业体系，加快发展新型文化业态，支持文化企业做精做特做大做强，叫响大别山（六安）品牌。深入挖掘文化资源，加大文艺创作力度，打造更多群众喜闻乐见、体现六安地域特色的精品力作。抓好四级公共文化设施网络体系建设，加快推进公共文化服务标准化、均等化，让文化发展成果更好惠及全市人民。
　　第四，全力构建和谐社会。坚持以人为本，深入实施民生工程，持续加大对贫困地区和困难群体帮扶力度。健全统筹城乡的就业服务体系，加强社保基金的征缴、发放和管理，不断提升社会保障水平。优先发展教育事业，全面推进素质教育，促进各级各类教育协调发展。深化医药卫生体制改革，加快健全全民医保体系，努力提高人民健康水平。逐步落实完善生育政策，稳定低生育水平，统筹解决人口问题。加强基层社会治理和服务体系建设，改进社会治理方式，完善治安防控体系，努力把六安建设成为全省最和谐稳定的地区之一。
　　第五，加强生态文明建设。牢固树立“绿水青山就是金山银山”的理念，坚守生态红线，坚持绿色发展，着力打造山青、水净、天蓝、气爽的人居环境。认真实施《安徽省主体功能区规划》，进一步处理好保护与开发的关系。编制实施生态文明建设规划，坚持工程治理与自然修复相结合，加大沿淮、沿湖、沿库湿地恢复治理力度。加强水源地保护和水土保持，深入推进江淮分水岭生态脆弱区治理和大别山区水源涵养林二期建设，继续实施“森林增长工程”，提高全市森林覆盖率，增强大别山区生态环境承载力。进一步强化土地节约集约利用，推进矿产资源有序开发、合理利用。大力推进循环经济示范园区和企业建设，培育壮大新能源、新材料等节能环保产业。强力推进节能减排，加强预警监管和执法，确保完成省下达任务。深入开展生态文明宣传教育活动，大力弘扬生态文化。
　　三、扎实做好2014年工作
　　2014年是新一届政府任期的第一年，也是奋力冲刺“十二五”规划的关键一年，做好今年的经济社会发展工作具有十分重要的意义。今年全市主要预期目标是：生产总值增长10%，财政收入增长11%，固定资产投资增长18%，规模工业增加值增长13%，社会消费品零售总额增长13%，城镇居民人均可支配收入增长10%，农民人均纯收入增长12%，城镇登记失业率控制在4.2%以内，人口自然增长率控制在7.5‰以内，完成年度节能减排和大气污染防治目标任务。重点抓好以下八个方面工作：
　　（一）促进经济平稳较快发展
　　推动项目建设提质提效。紧紧围绕国家宏观政策导向，以市重点项目“253工程”为抓手，着力增加有效投入，优化投资结构，带动产业升级。加强重大项目储备库建设，拓展项目谋划广度和深度。深入研究国家投资动向和财政资金扶持重点，积极争取项目资金。继续完善分级分层调度机制，建立健全项目考核机制，强化项目实施的指导、督促、协调和推进，加快首矿大昌300万吨钢铁、华电六安电厂二期、华润啤酒/水饮品双百万吨、武钢霍邱冶金工业园、舒城电子产业园、叶集家居产业园、大别山旅游扶贫快速通道、合六南通道一期、济祁高速六安段、六舒三路等重点项目建设，推进应流集团高端装备模块化生产、众兴集团云母深加工、博微长安产业园、六安火车站改扩建、金寨抽水蓄能电站、商杭高铁寿县站等项目尽早开工。
　　大力促进消费和进出口。完善城乡流通体系，积极引导城乡居民消费。全面实施“五位一体便民服务工程”，加快发展农村连锁直营便民超市，切实保障城乡消费安全便利。提升中心城区商贸服务业水平，大力推动城市综合体和商贸特色街区建设，加快标准化菜市场及社区连锁店改造与建设。促进产销衔接，开拓六安名优特产品市场。扶持壮大外贸企业队伍，鼓励企业大力开拓新兴市场，形成新的外贸增长点，争取获批出口加工区、保税仓库，力争进出口总额增长12%。
　　提高招商引资水平。制定产业指导目录，着力开展产业招商。拓宽招商领域，引导社会资本投资土地一级开发、标准化厂房、城市公用设施及社会公共事业。谋划重点招商项目，紧盯央企、知名民企和行业领军企业，开展一对一精准招商。改进驻外招商，着力加强与有关商会、协会、开发园区、企业和科研院所的联系与合作。严把项目准入关，努力提高项目投资强度和税收贡献率。建立健全招商项目推进机制，坚持首问负责制、限时办结制和责任追究制。力争到位内资和外商直接投资分别增长15%。
　　加快地方金融体系建设。深入开展“金融普惠年”活动，推动金融产品与服务创新，优化信贷结构，确保实现新增贷款规模增速不低于存款规模增速、制造业贷款增速不低于各项贷款平均增速、小微企业和“三农”贷款增速不低于当年各项贷款平均增速、贷款增量不低于上年同期水平。深入推进农村金融综合改革，全面完成全市农村合作金融机构改革任务。争取域外金融机构在我市设立分支机构，鼓励境内外资本在我市投资参股、直接设立融资租赁、股权投资、创业投资等金融类企业。积极培育企业上市资源，加快推动中小企业在“新三板”和区域性股权托管交易中心挂牌，力争5家以上企业上市或挂牌，全年直接融资20亿元。加强金融风险防控，优化金融生态环境。
　　（二）全力推进新型工业化
　　推进集约发展。进一步完善开发园区总体规划和产业发展规划，明晰产业定位，突出主导产业，优化空间布局，彰显各自特色，实现错位发展。高标准推进园区基础设施建设，完善功能配套，提升承载能力。推进省级开发区以“一区多园”、“托管区”、合作共建等形式整合周边乡镇工业集中区。加强园区土地节约集约利用，严格执行产业规划、环境保护、投资强度、税收贡献等项目准入标准，优先保证重点急需项目用地；建立项目用地供应双向约束机制，强化土地批后监管；加大闲置土地处置力度，加快建设标准化厂房和科技孵化器。推进园区循环化改造，培育循环经济示范园区。
　　扩大有效投入。启动实施千亿工业项目投资计划，加大重点项目推进力度，促进在建项目加快建设。设立财政专项资金，扶持骨干企业项目建设和小微企业发展，鼓励优势企业通过产业链整合、兼并重组、扩大产能、股改上市等途径做大做强，力争全年工业投资达到500亿元，增长25%；规模以上企业总数达到1000户，产值10亿元以上企业达到22户。
　　加大技术创新。启动实施工业转型升级五年行动计划，大力推动企业实施技术创新，引导龙头企业建立以研发中心、技术中心为重点的创新平台，鼓励中小企业以购买新技术、研发新产品为重点，提升创新能力。立足融合发展，建设一批省市级两化融合示范基地，推动传统产业新兴化和新兴产业规模化。以推行现场管理、加强质量标准建设、建立现代企业制度为重点，推进企业管理提升，提高管理水平。
强化分类指导。坚持扶优扶强，完善要素配置，集中土地、资金等要素，优先向重大优质工业项目倾斜。完善创业服务体系，加强企业公共服务平台和创业基地建设，促进中小企业发展。强化经济运行调度，确保生产要素衔接，帮助企业开拓国内外市场。充分利用好各类资本市场，鼓励优质企业开展直接融资。
　　（三）进一步加快农业现代化
　　提高农业综合生产能力。深入推进粮食生产“三大行动”和高产创建示范片建设，引导主要农产品向优势产区集中。深入实施百万亩油茶产业发展行动计划和茶产业振兴工程，大力发展绿色农业、高效农业、订单农业和休闲农业，加快推进现代农业示范区建设。组织开展畜禽、水产良种体系建设和健康养殖，大力开发皖西白鹅、高山蔬菜、水库有机鱼等地方优势品种资源，培育发展大别山特色农产品市场。合理布局农产品加工园区，开展产业链上下游分工合作，提升农产品精深加工能力和核心竞争力。加快粮食产业化发展，强化粮食流通市场监管。
　　培育新型农业经营主体。着力构建以专业大户、家庭农场、农民合作社、龙头企业为骨干，其他组织形式为补充的新型农业经营体系。加大对家庭农场的定向扶持，重点支持种养大户成立家庭农场。促进农民合作社持续健康发展，积极发展股份合作、土地合作等新型合作模式。深入开展科技特派员创业行动，大力培育和发展科技专家大院创新模式。按照基地型、集团化、链条式思路培育壮大龙头企业，力争农产品加工产值达到820亿元，增长15%，亿元以上龙头企业达120家。加快推进“中国绿谷”农产品物流园、茶叶综合体等重点招商项目。
　　强化农产品质量安全监管。支持发展品牌农业，新增 “三品一标”认证数量40个。推进标准化生产示范基地建设，组织开展国家级蔬菜、茶叶、水果、蚕桑标准园和畜禽水产养殖标准化示范场创建活动。加强农产品从田间到餐桌全过程监管，建立健全农产品质量可追溯制度，确保人民群众“舌尖上的安全”。
　　提升农村基础设施建设水平。继续推进农业综合开发、农村土地整治和高标准农田建设，组织实施国家千亿斤增粮规划、江淮分水岭地区综合治理和巩固退耕还林成果建设项目。解决50万农村人口饮水安全问题，实施7条中小河流治理，完成90座小II型水库除险加固任务，治理水土流失面积110平方公里。积极推进新一轮治淮工程建设，大力实施“八小”农田水利工程。加强农村气象灾害防御体系建设，加快农村电网和通讯设施提标扩面进程，升级改造县乡公路300公里，改造农村公路危桥90座。
　　（四）大力发展新型城镇化
　　强化规划引领与管控。开展城市发展战略规划研究，合理确定城镇开发边界和强度，优化城镇化布局和形态。完善规划体系，健全控制性详细规划和专业规划，加强重点区域城市设计，注重生态保护，传承城市文化，塑造城市特色，提升城市品位。加快推进寿县、叶集新一轮城市总体规划修编工作，完成10个中心镇总规调整。强化规划集中统一管理，加大巡查执法力度，依法查处违法行为。
　　强力推进中心城市建设与管理。启动新一轮中心城市建设重点工程四年实施计划，今年计划政府性投资49亿元。加快东部产业新城和西部生态新区建设，完善功能配套，提升承载能力。强化市区土地集中管理，统一土地储备、供应、登记和执法，继续抓好土地出让金征收管理。进一步完善政府投资项目招投标制度，着力解决施工单位“挂靠”问题，减少服务类招标“流标”现象，提高招投标工作效率。巩固省级卫生城市创建成果，深入开展“五城同创”和国家森林城市创建工作。进一步理顺城管执法体制，推动重心下移，调动各级各方面积极性，提高城市管理精细化水平。组织开展市容市貌、超载超限、渣土运输等专项整治。引导房地产业健康发展，提升物业管理水平。
　　加快县城和中心镇、美好乡村建设。加强分类指导，研究出台支持政策，推进各县县城和叶集城区建设扩容提质。加强城镇地下管网、防洪排涝、道路交通、垃圾与污水处理等基础设施建设，抓好城镇绿化美化。继续推进扩权强镇试点，抓好50个重点镇建设。开展“三线三边一点一地” 城乡环境综合治理，进一步改善城乡面貌特别是农村人居环境。实施美好乡村建设第二批中心村建设和自然村改造，加大资金整合和投入力度，建立健全美好乡村投入、管理、服务等长效机制。突出主导产业培育，促进农民持续增收。出台支持村级集体经济发展相关政策，提高村级发展能力。
　　全方位开展区域合作。加强大别山区域城市合作，争取国家启动实施大别山革命老区振兴发展规划。加大合肥经济圈合作力度，加快构建合六产业走廊，优化“一轴两翼”产业布局；拓展寿县、霍邱与合肥市结对合作领域，积极参与合肥空港经济示范区建设，落实舒城与合肥市合作发展重点事项，推进市示范园区与肥西县加强产业合作和基础设施共建。推动金寨、叶集、霍邱基础设施共建、产业互动发展。
　　（五）深化重点领域和关键环节改革
　　加快行政体制改革。积极稳妥推进新一轮政府机构改革，完成工商、质检和食品药品监管体制改革任务。继续深化行政审批制度改革，优化审批流程，推进政务服务标准化。加快推进事业单位分类改革，进一步规范市直单位编外聘用人员管理。加快实施政社分开，推进社会组织明确权责、依法自治、发挥作用，推动行业协会商会和中介服务组织与行政机关真正脱钩。
　　深化农村综合改革。坚持和完善农村基本经营制度，启动金寨县、金安区农村综合改革示范试点。加快推进土地承包经营权确权登记发证工作，健全土地流转激励机制，引导土地向新型农业经营主体流转。构建农村集体资金资产资源管理新机制，创新农村公共基础设施建设和管理机制。稳步开展农村土地承包经营权、宅基地使用权抵押贷款试点。积极稳妥推进国有林场改革试点。
　　推进财税改革。坚持事权和支出责任相适应原则，完善市区财政体制。改进预算管理制度，完善政府预算体系，实施全口径预算管理，稳步推进预决算和“三公”经费信息公开。完善税收管理制度，进一步发挥综合治税平台作用，提高征管水平。继续做好“营改增”扩围试点工作，跟踪房地产税改革。规范政府融资平台运行机制，深化土地收储和投融资管理体制改革。加强政府性债务管理，做好债务风险防控和化解工作。
　　促进市场化改革。全面推进市属国有企业改制工作，妥善解决遗留问题。深化行政事业单位国有资产和国有资源管理改革，强化集中管理与统一运营。推进城市供水、污水处理市场化，引进战略投资者，提高运营水平。实施城市公交车、出租车运营体制改革。积极探索政府购买服务。进一步健全政策措施，放宽市场准入，降低创业门槛，推动民营经济持续快速发展。
　　（六）加快转型发展步伐
　　增强创新驱动发展能力。加快推进国家技术创新工程试点，新培育创新型企业6家、知识产权优势企业8家、工程技术研究中心10家。进一步落实与高校院所战略合作协议，建立产学研协同创新机制，筹建六安大学科技园。大力发展高新技术产业，实现产值300亿元，新认定国家高新技术企业8家。强化知识产权的创造、运用和保护，实现发明专利申请800件以上、授权100件以上。研究编制重点新兴产业发展规划，组织实施战略性新兴产业“倍增工程”，力争全年战略性新兴产业产值达到150亿元，增长14%。
　　提升现代服务业。组织实施服务业发展规划，重点培育科技研发、金融保险、物流会展、中介服务、总部经济等生产性服务业，大力发展商业酒店、休闲娱乐、社区服务、健康养老等生活性服务业。加快推进吉宝冷链物流园、裕安城南物流中心建设，吸引服务外包企业入驻。推进电子商务与快递物流融合发展，建设电商快递物流配送园。做好旅游业发展总体规划和红色旅游规划修编，加快五大旅游区建设，加强旅游资源整合和景区景点开发力度，大力发展红色旅游和乡村旅游，打造大别山旅游品牌。
　　着力推进文化强市建设。坚持用社会主义核心价值观引领社会风尚、凝聚社会共识，筑牢实现中国梦的思想基础。扎实做好道德模范评选表彰活动，广泛开展志愿服务。健全公共文化服务财政保障机制，加快构建现代公共文化服务体系，着力推进市图书馆、文化馆等重点文化项目建设。组织开展重要革命纪念场馆陈列布展升级改造，加快国家级爱国主义教育基地建设。积极推进农民文化乐园建设，丰富群众业余文化生活。大力发展文化产业，加快悠然蓝溪、保利文化产业园、皋陶文化园等重点项目建设，推进文化产业特色化、品牌化发展。做好文物保护工作，加强非物质文化遗产保护和传承。
　　进一步加强生态建设。积极创建国家生态文明先行示范区，加强重点流域水污染防治，加快淠河总干沿线和瓦埠湖环境综合治理。高度重视大气污染防治，加强城区空气质量监测，形成PM2.5监测能力，积极防控重污染天气。继续加强重点企业和减排项目监管，加大环保执法力度，严厉打击违法行为，确保完成年度节能减排任务。落实最严格的耕地保护制度和水资源管理制度，加强水源地保护和水土保持，强力推进“森林增长工程”。大力发展循环经济，提高资源利用效率。继续深入开展农村环境连片整治。
　　（七）全力保障和改善民生
　　巩固提升民生工程。在认真实施省定民生工程基础上，积极推进农副产品平价商店建设、老旧小区改造、城区报警与视频监控系统建设、农贸市场改造，着力解决群众普遍关注、反映强烈的突出问题，努力让更多群众满意。加强民生工程项目绩效管理，建立分类管养机制，确保项目效益充分发挥。开展已建成民生工程项目产权确认登记，实现国有资产保值增值。
　　做好就业和社会保障工作。完善支持和促进就业的政策措施，加强职业技能培训，多渠道开发就业岗位，统筹推进重点人群就业，实现城镇新增就业3.1万人。进一步巩固城乡居民社会养老保险制度全覆盖成果，提高社会保险统筹层次。完善城镇职工医疗、居民基本医疗、新型农村合作医疗和大病救助制度，着力构建工伤预防、补偿、康复三位一体制度体系。高度重视养老问题，积极构建以居家养老为基础、社区养老为依托、机构养老为支撑的社会养老服务体系。继续推进保障性安居工程建设，完善建设、分配、管理和退出机制，加快棚户区改造步伐。
　　扎实推进扶贫攻坚。认真实施《大别山片区区域发展与扶贫攻坚规划（2011-2020年）》，创新大扶贫模式，实施精准扶贫，大力推进行业扶贫、专项扶贫和社会扶贫。加大产业化扶贫力度，培育发展特色产业，增强自身造血功能。加强统筹协调，整合项目资金，“整村推进”180个贫困村。继续实施“雨露计划”和农村教育补贴试点工作。强力推进金寨“5+1”项目，充分发挥示范引领、辐射带动作用。
　　（八）努力构建和谐社会
　　统筹发展各项社会事业。大力实施义务教育标准化建设，启动六安城区义务教育阶段公办学校建设三年行动计划，推进义务教育就近免试入学。优化城区教育布局，加快六安一中东校区、二中西校区和青少年综合实践基地建设。充分发挥大别山职教集团引领作用，加快构建现代职业教育体系。创新高校人才培养机制，支持皖西学院创建应用型示范本科高校。加强教师队伍建设，提升师德水平和业务能力。进一步健全城乡医疗卫生服务体系，提高基层医疗卫生服务能力，加快市人民医院西院区和市中医院二期工程建设。深入开展爱国卫生运动，落实重大疾病防控和慢性病干预措施。继续深化医药卫生体制改革，鼓励社会办医。广泛开展群众性体育活动，完善全民健身体系。切实加强流动人口计生服务管理和出生人口性别比综合治理，加快人口计生转型发展。继续做好第三次全国经济普查，规范企业、乡镇统计工作。推进残疾人服务和保障体系建设，切实保障残疾人权益。推动广播影视、新闻出版、地方志、气象、地震、老龄、档案、对台、外事侨务等事业发展。
　　创新社会治理。完善基层社会治理和服务体系，提高社区（村）网格化服务治理水平。学习“枫桥经验”，进一步畅通群众诉求渠道，积极推广网上信访，推进诉访分离，规范信访秩序。深入推进“六五”普法，加强法律援助和社区矫正安置帮教工作。加大依法管理网络力度，引导社会舆论，确保网络和信息安全。认真贯彻落实党的民族政策，依法管理宗教事务。进一步推动平安六安建设，着力推进农村平安服务网建设等十项重点工程，有效防范和严厉惩治各类违法犯罪活动，确保社会安定有序、人民安居乐业。
　　加强公共安全防控。健全应急管理体制机制，做好防灾减灾工作，不断提升应对突发事件的预防预警和处置能力。改革和完善食品药品安全监管体系，深入开展专项整治和综合治理，保障人民群众食品药品安全。严格落实安全生产责任制，强化地质灾害、矿业生产、道路交通、建筑施工等重点领域的安全监管，加大烟花爆竹生产企业退出力度，有效预防和遏制较大事故发生。强化国防教育，做好民兵、兵役、双拥、优抚和复转军人安置工作；推动军民融合发展，加强国防动员基础建设，提高后备力量建设质量。
　　各位代表，事业任重道远，形势催人奋进。我们要以改革创新的精神全面加强政府自身建设，一招不虚、一步不让抓发展，踏石留印、抓铁有痕促落实，努力建设职能科学、结构优化、廉洁高效、人民满意的服务型政府。我们要牢记为民宗旨。坚持执政为民，深入开展群众路线教育实践活动，始终做到为人民服务、对人民负责、受人民监督、让人民满意。尊重群众首创精神，走进一线察民情，深入基层接地气，提升群众工作能力。牢固树立责任意识和争先进位意识，加强压力传递，层层落实责任，勇于负责，敢于担当。完善发展成果考核评价体系，更加重视生态保护、居民收入、社会保障、安全稳定。我们要加快职能转变。正确处理政府与市场、政府与社会的关系，解决好政府越位、缺位问题，着力在创造良好发展环境、提供优质公共服务、维护社会公平正义等方面下功夫。坚持效率优先，进一步健全重点工程建设、征迁、城管大联勤、治超、基本建设项目联合审批、招商引资项目推进、市场管理、国有资产管理、政府融资、税收征管协作等工作机制。持续推进机关效能建设，加大“庸、懒、散”整治力度，完善绩效评估，推动政府工作提速提质提效。我们要严格依法行政。进一步完善行政决策程序，坚持把公众参与、专家论证、风险评估、合法性审查、集体讨论贯穿于重大行政决策全过程。大力推进政务公开，严格依照法定权限和程序行使权力、履行职责，让权力在阳光下运行。加强行政执法监督和行政复议监督，促进规范执法。进一步加大对重点领域、重点部门、重点项目的行政监察和审计监督，加强对党政主要领导干部和国有企业领导人员经济责任审计，切实抓好审计结果运用。自觉接受人大监督和政协民主监督，广泛听取各民主党派、工商联和无党派人士的意见和建议，充分发挥工会、共青团、妇联等人民团体的桥梁纽带作用，高度重视舆论监督和社会公众监督。我们要强化廉政建设。严格落实党风廉政建设责任制，全面推进惩治和预防腐败体系建设，坚决反对“四风”。进一步加强公共资源交易平台和电子招投标平台建设，强化对招投标、政府采购、土地出让、产权交易等重点领域和关键环节的监管，严厉查处各种违法违纪案件。切实改进文风会风，大力精简会议文件，坚决控制各类检查评比，把更多的时间和精力用在谋发展、抓落实、办实事上。厉行勤俭节约，严控“三公”经费支出、楼堂馆所建设和表彰庆典活动，切实降低行政成本，把有限的财力和资源用在推动发展、改善民生上，以为民、务实、清廉的形象赢得人民群众的拥护和支持。
　　各位代表，站在新的起点，面对新的挑战，我们倍感使命光荣、责任重大。让我们在中共六安市委的坚强领导下，弘扬红色精神，致力绿色发展，放飞金色梦想，万众一心，励精图治，扎实推进幸福六安建设，为全面建成小康社会而努力奋斗！
